# The role of microRNAs in ferroptosis

**DOI:** 10.3389/fmolb.2022.1003045

**Published:** 2022-10-12

**Authors:** Liqing Guo, Qingkun Zhang, Yuehui Liu

**Affiliations:** ^1^ Department of Otolaryngology, The Second Affiliated Hospital of Nanchang University, NanChang, China; ^2^ Jiangxi Province Key Laboratory of Molecular Medicine, Nanchang, China

**Keywords:** ferroptosis, lipid peroxidation, microRNA, cardiomyopathy, tumor, neuronal injury disorder, ischemia perfusion disorder

## Abstract

Ferroptosis is a newly discovered type of programmed cell death, which is closely related to the imbalance of iron metabolism and oxidative stress. Ferroptosis has become an important research topic in the fields of cardiomyopathy, tumors, neuronal injury disorders, and ischemia perfusion disorders. As an important part of non-coding RNA, microRNAs regulate various metabolic pathways in the human body at the post-transcriptional level and play a crucial role in the occurrence and development of many diseases. The present review introduces the mechanisms of ferroptosis and describes the relevant pathways by which microRNAs affect cardiomyopathy, tumors, neuronal injury disorders and ischemia perfusion disorders through regulating ferroptosis. In addition, it provides important insights into ferroptosis-related microRNAs, aiming to uncover new methods for treatment of the above diseases, and discusses new ideas for the implementation of possible microRNA-based ferroptosis-targeted therapies in the future.

## Introduction

Ferroptosis is a newly discovered programmed cell death mechanism with characteristics that are different from those of autophagy, apoptosis and necrosis. Several studies have demonstrated that ferroptosis is involved in the occurrence and development of cardiomyopathy, tumors, neuronal injury disorders, and ischemia perfusion disorders (Masaldan et al., 2019; Jiang et al., 2021; Van Coillie et al., 2022). MicroRNAs are a class of endogenous non-coding small RNA molecules, generally 21–25 nucleotides in length, which regulate various metabolic pathways in the human body at the post-transcriptional and translational levels, including regulation of tumor cell growth and induction of chemotherapy resistance (Ambros, 2001). Noncoding RNAs play a significant role in ferroptosis of various cells (Zhang X. et al., 2020; Zhi et al., 2021; Zuo et al., 2022). Previous reviews have mainly focused on the role of noncoding RNAs in cancer through regulating ferroptosis (Valashedi et al., 2022), but the role of microRNAs in other diseases through regulating ferroptosis has not been summarized.

The present review first discusses the mechanisms of ferroptosis and then summarizes the diseases and related pathways regulated by microRNAs through regulating ferroptosis. MicroRNAs have great potential as therapeutic targets. This report provides reference information for more in-depth studies of microRNAs in the field of ferroptosis.

### Overview of ferroptosis

In 2012, Dixon et al. first used the concept of ferroptosis to describe the mode of cell death, which is caused by the accumulation of lipid peroxides (Dixon et al., 2012).

Ferroptosis is an iron-dependent mode of cell death that involves lipid reactive oxygen species (ROS) accumulation (Stockwell et al., 2017). The mechanism of its occurrence has been partially uncovered (Jhelum et al., 2020). First, the overloaded iron ions generate a lot of free hydroxyl groups *via* the Fenton reaction. Iron is a vital trace element involved in many physiological processes in the human body. Excess iron, however, increases the oxidative sensitivity of cells (Chen et al., 2019). Cells take up Fe^3+^ primarily through the transferrin receptor protein 1 complex (Gao et al., 2015). The six-transmembrane epithelial antigen of the prostate 3 (Steap3) reduces intracellular Fe^3+^ to Fe^2+^. Ferroportin (FPN), also known as solute carrier family 40 member 1 (SLC40A1), is involved in the regulation of iron balance by expelling Fe^2+^ out of the cells (Geng et al., 2018). Overexpression of ferritin heavy chain1 (FTH1) inhibits erastin-induced ferroptosis by regulating Fe^2+^ (Gao et al., 2016; Hou et al., 2016).

Next, lipid peroxidation induction leads to lipid metabolism disorders, and the accumulation of lipid oxides and ROS is responsible for ferroptosis. Fe^2+^ overload causes a series of oxidative stress reactions in the cell, resulting in the destruction of the nucleus, membrane, organelles, and proteins. Esterified polyunsaturated fatty acids (PUFAs) are the most common substrates in lipid peroxidation (Kagan et al., 2017). Acyl-CoA synthetase long-chain family member 4 (ACSL4) is involved in the biosynthesis and reassembly of PUFAs on the cell membrane (Chen et al., 2021b). Lysophosphatidylcholine acyltransferase 3 then mediates PUFA activation to induce ferroptosis (Doll et al., 2017).

Finally, System Xc^−^ - glutathione - glutathione peroxidase 4 (Xc^−^-GSH-GPX4) is considered a crucial antioxidant axis in ferroptosis. Xc^−^ complex is a cysteine-glutamate reverse transporter composed of two-subunit solute carrier family 7 member 11 (SLC7A11/xCT) along with solute carrier family 3 member 2 (SLC3A2) (Koppula et al., 2018). The main function of Xc^−^ is to regulate the transport balance of glutamate and cysteine and participate in the synthesis of GSH (Koppula et al., 2021). GSH is a cofactor for GPX4. It protects cells from oxidative damage. GPX4 is a selenoprotein that neutralizes toxic lipid peroxides and inhibits ferroptosis (Forcina and Dixon, 2019; Weaver and Skouta, 2022). GPX4 inactivation is a pivotal condition for the occurrence of ferroptosis (Jhelum et al., 2020). Large amounts of GSH are depleted, resulting in decreased glutathione peroxidase 4 (GPX4) activity. Certain intracellular components, including oncoprotein activating transcription factor 4 (ATF4), nuclear factor erythroid 2-like factor 2 (NFE2L2/NRF2), and Beclin-1, can also regulate iron by affecting systems Xc^−^ and GSH death level (Habib et al., 2015; Song et al., 2018). In addition, the classic tumor suppressor gene P53 affects the occurrence and development of various diseases, such as tumors, by regulating GPX4-dependent and GPX4-independent ferroptosis pathways (Liu and Gu, 2022).

At present, numerous genetic hallmarks and protein hallmarks of ferroptosis are available for detection, but their specificity remains limited (Chen et al., 2021a).

Other auxiliary evidence, such as detection of cell activity, iron levels, GSH levels in cells and tissues, ROS and ROS product content, malondialdehyde (MDA), and mitochondrial membrane potential (MMP) is also often used to demonstrate the occurrence of ferroptosis. Moreover, changes in cell morphology, particularly mitochondrial morphology, observed under transmission electron microscopy, are the main features that distinguish ferroptosis from other forms of programmed cell death, such as apoptosis, autophagy, and necrosis. Mitochondrial shrinkage, high membrane density, diminished or absent cristae, and exterior membrane rupture are common in cells undergoing ferroptosis (Agmon and Stockwell, 2017; Stockwell, 2022).

More research on ferroptosis has continued to accumulate over the last few years. Ferroptosis has been shown to take part in the development of various diseases, such as tumors, cardiovascular diseases, and autoimmune diseases (Nguyen et al., 2020; Guo et al., 2022; Lai et al., 2022). Next, the role of microRNAs was explored in ferroptosis separately from microRNA-involved cardiomyopathy, tumors, nervous system diseases, and ischemia perfusion disorders.

### The role of microRNA in cardiomyopathy *via* regulating ferroptosis

As mentioned earlier, GPX4 is critical for the regulation of ferroptosis (Ursini et al., 2022). Zhuang et al. used an ischemia-reperfusion (I/R) rat model and cardiac fibrosis cell model induced by angiotensin II to determine that miR-375–3p promotes ferroptosis and accelerates cardiac fibrosis by inhibiting GPX4 (Zhuang et al., 2022). Fan et al. have showed that inhibition of miR-15a-5p decreases ferroptosis through GPX4 and thus alleviates myocardial injury in acute myocardial infarction (Fan et al., 2021). Inhibition of miR-1224 has also been found to alleviate hypoxia/reoxygenation myocardial injury by upregulating GPX4 (Li G. et al., 2021). GPX4 is a critical target in ferroptosis. MiR-375-3p, miR-15a-5p or miR-1224 inhibition may protect cardiomyocytes and alleviate myocardial injury by increasing GPX4 and decreasing ferroptosis. As a part of the Xc^−^ transporter, SLC7A11 also plays a significant role in the antioxidant system (Jyotsana et al., 2022). The study of Liu et al. inhibited cardiac fibroblast-derived exon-miR-23a-3p by using the exosome inhibitor GW4869, and inhibition of miR-23a-3p resulted in upregulation of SLC7A11, thereby reducing ferroptosis in H9c2 cardiomyocytes and preventing continued development of atrial flutter (Liu D. et al., 2022). It suggests that miR-23a-3p inhibition increases intracellular cystine and GSH levels, thereby neutralizing ROS and treating atrial fibrillation. Glutaminase 2 (GLS2) can cause ROS accumulation through mitochondria by accelerating glutamate formation (Matés et al., 2020). After myocardial infarction, cardiomyocytes often undergo cell death and pathological remodeling, which easily lead to heart failure (Duan, 2020). Zhou et al. have suggested that miR-190a-5p inhibits cardiomyocyte ferroptosis by inhibiting GLS2 and decreases the levels of ROS, MDA, and Fe^2+^ in H9c2 cells, thus playing a protective role in myocardial infarction (Zhou et al., 2021). MiR-190a-5p reduces lipid peroxidation by inhibiting GLS2 mRNA, thereby inhibiting ferroptosis and lowering the risk of myocardial infarction. Autophagy related protein 5 (ATG5) has been suggested to inhibit ferroptosis by inhibiting autophagy (Liang et al., 2022). Tang et al. have found that overexpression of miR-30d promotes ferroptosis after myocardial infarction by targeting ATG5, thus inhibiting autophagy in cardiomyocytes (Tang et al., 2020). Therefore, crosstalk may exist between ferroptosis and autophagy. The incidence of cardiovascular disease is high worldwide. Decreasing cardiomyocyte death and repairing damaged cardiac tissue are urgent clinical needs (Xu S. et al., 2021). Ferroptosis decreases reduces the overall cardioprotective effect of ischemia/reperfusion (I/R) injury. Ferroptosis suppression decreases inflammation and limits the extent of left ventricular remodeling after I/R injury (Komai et al., 2022).

Common clinical cardiovascular diseases include atherosclerosis, myocardial infarction, heart failure, and arrhythmia. Ferroptosis plays a vital role in the development of cardiovascular disease (Guo et al., 2022). The mechanism of microRNA in ferroptosis occurs through regulation of the antioxidant system and lipid oxidation. Most existing studies have shown that microRNA overexpression damages cardiomyocytes by promoting ferroptosis. However, because microRNAs have diverse functions, further research is needed to reveal more details. Table 1 shows microRNAs that affect cardiomyopathy through regulating ferroptosis.

**TABLE 1:** The role of microRNA in cardiomyopathy *via* regulating ferroptosis.

microRNA name	Target gene	Cell model	Disease name	Effect on ferroptois
miR-23a-3p	SLC7A11	H9c2	Atrial fibrillation	Promote ferroptosis
miR-375–3p	GPX4	Cardiac fibroblasts	Cardiac fibrosis	Promote ferroptosis
miR-190a-5p	GLS2	H9c2	Myocardial infarction	Suppress ferroptosis
miR-30d	ATG5	H9c2	Myocardial infarction	Promote ferroptosis
miR-1224	GPX4	H9c2	Myocardial H/R injury	Promote ferroptosis
miR-15a-5p	GPX4	HL-1	Myocardial infarction	Promote ferroptosis

### The role of microRNA in tumors *via* regulating ferroptosis

GPX4 remains an important target of microRNAs in tumor diseases (Gao et al., 2022). The experiments by Xu et al. showed that miR-15a overexpression can inhibit cell proliferation, increase the release of lactate dehydrogenase, MDA, Fe^2+^, and ROS, and then destroy MMP by inhibiting GPX4 in prostate cancer cells (Xu P. et al., 2022). In colorectal cancer cells, Liu et al. reported that miR-15a-3p overexpression can also promote ferroptosis by inhibiting GPX4 (Liu L. et al., 2022). Xu et al. found that miR-1287–5p still targets GPX4 to promote ferroptosis in osteosarcoma cells (Xu Z. et al., 2021). Deng et al. study suggested that miR-324–3p targets inhibition of GPX4, promotes ferroptosis in lung adenocarcinoma cells, and reverses their resistance to cisplatin (Deng et al., 2021). These microRNAs directly bound to the 3′-UTR of GPX4 mRNA and inhibited its expression, causing ROS accumulation in tumor cells and acting as tumor suppressor genes. Inhibition of the ATF4-HSPA5-GPX4 pathway reduces GPX4 levels and induces ferroptosis. Loss of ATF4 leads to increased ferroptosis (Chen et al., 2017; Zhu et al., 2017). The study by Bai et al. reported that miR-214–3p targets ATF4 to promote ferroptosis in hepatoma cells (Bai et al., 2020). Gomaa et al. found that overexpression of miR-4715–3p can inhibit aurora kinase A (AURKA) and GPX4, inducing ferroptosis in upper gastrointestinal adenocarcinoma cells (Gomaa et al., 2019). GPX4 content is a key factor in tumor ferroptosis, and these microRNAs have been shown to promote ferroptosis in various tumor cells by inhibiting target genes involved in GPX4 synthesis. Ferroptosis suppressor protein 1 (FSP1) exerts antioxidant effects parallel to GPX4, and FSP1-CoQ10-NAD(P)H is another pathway that inhibits ferroptosis (Bersuker et al., 2019; Doll et al., 2019). One experiment showed that exosomal miR-4443 targets methyltransferase 3 (METT3) to regulate FSP1 expression, thereby inhibiting ferroptosis in non-small cell lung cancer (Song et al., 2021). In the absence of GPX4, FSP1 can be used as an oxidoreductase to inhibit ROS and alleviate ferroptosis.

SLC7A11 is widely expressed in various tumor tissues, and plays a significant role in inhibiting ferroptosis during the occurrence and development of tumors by controlling cysteine transport (Tang et al., 2022). Sun et al. found that the miR-34c-3p/SLC7A11 axis can potentiate erastin-induced ferroptosis in oral squamous cell carcinoma (Sun et al., 2022). Yadav et al. reported that overexpression of miR-5096 can promote ferroptosis by targeting SLC7A11 and inhibit breast cancer cell growth (Yadav et al., 2021). The study by Ni et al. showed that miR-375/SLC7A11 inhibits gastric cancer stem cells by triggering ferroptosis. They believe that miR-375 can reduce the stemness of gastric cancer cells by inducing ferroptosis (Ni et al., 2021). SLC3A2, like SLC11A7, is an important functional subunit of the Xc^−^ system (Liu M. et al., 2022). Hu et al. found that exosomal miR-142–3p secreted by hepatocellular carcinoma cells targets SLC3A2 to promote ferroptosis in MI-type macrophages, thus, accelerating the development of hepatocellular carcinoma (Hu et al., 2022). The microRNAs mentioned above reduced GSH levels by regulating cystine transport into tumor cells by targeting SLC7A11 and SLC3A2. Dickkopf-related protein 1 (DKK1) inhibits the occurrence of ferroptosis and protects cells from ferroptosis by enhancing the expression of SLC7A11 (Wu M. et al., 2022). Liao et al. found that miR-130b-3p targets DKK1 to inhibit ferroptosis in melanoma cells (Liao et al., 2021). MicroRNAs essentially regulated tumor ferroptosis via the antioxidant system, whether by regulating GPX4, FSP1, or GSH.

ACSL4 can promote lipid peroxidation of PUFAs to promote ferroptosis, and is also an important target of many microRNAs (Chen et al., 2021b; Liu et al., 2021). Bao et al. demonstrated that miR-670–3p can inhibit ferroptosis in glioblastoma cells by targeting ACSL4. Furthermore, miR-670–3p inhibitor-treated U87MG and A172 cells increase chemosensitivity to temozolomide (Bao et al., 2021). The study by Ma et al. showed that miR-424–5p targeting ACSL4 negatively regulates ferroptosis in ovarian cancer cells (Ma et al., 2021). They inhibited ROS production from PUFAs by targeting ACSL4 mRNA, thus resulting in ferroptosis inhibition.

Solute carrier family 1 member 5 (SLC1A5) transports glutamine into the cell during ferroptosis, and may increase cell sensitivity to ferroptosis (Xu F. et al., 2022; Zhu D. et al., 2022). Luo et al. reported that overexpression of miR-137, which results in decreased glutamine and MDA levels, can inhibit ferroptosis in melanoma cells by targeting SLC1A5 (Luo et al., 2018). Aspartate aminotransaminase (GOT1) is an enzyme involved in glutamate metabolism, which catalyzes the production of α-ketoglutarate (Kremer et al., 2021). Zhang et al. have shown that overexpression of miR-9 inhibits ferroptosis in melanoma cells by directly binding to the 3′-UTR of GOT1 (Zhang et al., 2018). These findings suggest that miR-137 and miR-9 speed up the progression of melanoma by inhibiting ferroptosis through reducing lipid peroxidation.

Iron-responsive element-binding protein 2 (IREB2) is related to intracellular iron ion concentration, and suppression of IREB2 can inhibit the level of Fe^2+^, thus inhibiting ferroptosis (Li et al., 2022). Fan et al. showed that miR-19a inhibits ferroptosis in colorectal cancer cells HT29 by inhibiting IREB2 (Fan et al., 2022). Transferrin (TF) mediates Fe^2+^ entry into the cells, and TF blocking can reduce Fe^2+^ overload and inhibit ferroptosis (Pandrangi et al., 2022). Zheng et al. found that miR-545 inhibition can reduce ferroptosis in rectal cancer cells by regulating TF (Sixin Zheng, Lingling Hu, Qingwen Song et al., 2021). Intracellular Fe^2+^ can transport Fe^2+^ out of the cells *via* FPN1/SLC40A1. FPN participates in ferroptosis by regulating intracellular Fe^2+^ concentration (Hao et al., 2021; Wang et al., 2021; Pandrangi et al., 2022). Wei et al. reported that miR-302a-3p can strengthen ferroptosis in non-small cell lung cancer by targeting FPN (Wei et al., 2021). Zhu et al. showed that miR-4735–3p targets SLC40A1 to promote ferroptosis in clear cell renal cell carcinoma, thereby inhibiting tumor proliferation (Zhu C. et al., 2022). Ferroptosis is characterized by iron overload. Different microRNAs have different effects on iron metabolism target genes, resulting in ferroptosis regulation.

Tumor necrosis factor-α-induced protein 8 (TNFAIP8/TIPE) can participate in ferroptosis by inhibiting p53. One prior study showed that miR-539 overexpression can promote ferroptosis in colorectal cancer cells by inhibiting TIPE (Yang et al., 2021). Two studies by Tomita et al. demonstrated that miR-7-5p knockdown can reduce radioresistance in radioresistant cancer cells by regulating ferroptosis through changes in Fe^2+^ content (Tomita et al., 2019; Tomita et al., 2021). Tumor cells change their own microenvironment to achieve continuous proliferation (Xu et al., 2020), and microRNAs affect the proliferation of various tumor cells through the regulation of ferroptosis. The function of microRNA can be accomplished by the degradation or the translation inhibition of targeted mRNA after binding to the 3′-UTR region. However, this regulatory effect is complex, in that it presents two aspects of tumor-promoting and tumor-suppression. Table 2 lists microRNAs that affect tumors through regulating ferroptosis.

**TABLE 2:** The role of microRNA in tumor *via* regulating ferroptosis.

microRNA name	Target gene	Cell model	Disease name	Effect on ferroptois
miR-4735–3p	SLC40A1	786-O, A498	Cell Renal cellcarcinoma	promote ferroptosis
miR-19a	IREB2	HT29	Colorectal cancer	Suppress ferroptosis
miR-142–3p	SLC3A2	HepG2,THP-1	Hepatocellular carcinoma caused hepatitis B virus	promote ferroptosis
miR-34c-3p	SLC7A11	SCC-25,CAL-27	Oral squamous cell carcinoma	promote ferroptosis
miR-15a	GPX4	LNCAP	Prostate cancer	promote ferroptosis
miR-545	TF	HT-29,HCT-116	Colorectal cancer	Suppress ferroptosis
miR-15a-3p	GPX4	HCT-116,CaCo2, HT29, KM12	Colorectal cancer	promote ferroptosis
miR-539	TIPE	HCT-116	Colorectal cancer	promote ferroptosis
miR-5096	SLC7A11	MDA-MB-468,MDA-MB-453, BT-549, MDA-MB-231,SKBR-3, T-47D, MCF-7, ZR-75	Breast cancer	promote ferroptosis
miR-1287–5p	GPX4	SaOS2, U2OS	Osteosarcoma	promote ferroptosis
miR-7-5p	-	HeLa,SAS	*Cancer* radioresistance	Suppress ferroptosis
miR-670–3p	ACSL4	U87MG, A172	Glioblastoma	Suppress ferroptosis
miR-302a-3p	Ferroportin	A549,H358,H1299, H1650	Non-small cell lung cancer	promote ferroptosis
miR-130b-3p	DKK1	A375,G-361	Melanoma	Suppress ferroptosis
miR-375	SLC7A11	SGC-7901,BGC-823	Gastric cancer	promote ferroptosis
miR-424–5p	ACSL4	HO8910,SKOV3	Ovarian cancer	Suppress ferroptosis
miR-214–3p	ATF4	HepG2,Hep3B	Hepatocellular carcinoma	promote ferroptosis
miR-4715–3p	AURKA	OE33, MKN45	Upper gastrointestinal cancers	promote ferroptosis
miR-9	GOT1	A375, G-361	Melanomma	Suppress ferroptosis
miR-137	SLC1A5	A375, G-361	Melanomma	Suppress ferroptosis
miR-324–3p	GPX4	A549	Lung adenocarcinoma	promote ferroptosis
miR-4443	METT3	A549-R,A549S	Non-small cell lung carcinoma	Suppress ferroptosis

### The role of microRNA in neuronal injury disorder *via* regulating ferroptosis

FSP1 acts as a complementary antioxidant pathway and is also a target gene of microRNA-672–3p. Inhibition of miR-672–3p was reported to inhibit ferroptosis by upregulating FSP1, thereby promoting neural repair in spinal cord injury (Wang F. et al., 2022). The SLC7A11/GPX4 signaling pathway is a major antioxidant pathway in ferroptosis-induced nerve injury (Fu et al., 2022). Wang et al. reported that inhibition of miR-378a-3p reverses lead exposure-induced ferroptosis by targeting SLC7A11 in HT22 cells (Wang W. et al., 2022). The two microRNAs mentioned above induced ferroptosis by inhibiting key molecules in the antioxidant pathway. It suggests that blocking them can reduce nerve cell death and alleviate specific neuronal injury disorders.

BTB and CNC homology 1(Bach1) is an oxidative stress-responsive transcription factor in ferroptosis, which promotes it by inhibiting the antioxidant system (GSH-GPX4 and FSP1-CoQ10 pathways) (Nishizawa et al., 2022). Li et al. showed that miR-194-loaded mesenchymal exosomes can inhibit neurovascular endothelial cell ferroptosis by targeting Bach1, resulting in neuroprotection (Li X. et al., 2021). Prostaglandin peroxidase synthase-2(Ptgs2) is a regulatory gene in ferroptosis lipid oxidation (Macías-Rodríguez et al., 2020). Xiao et al. reported that overexpression of miR-212–5p overexpression can inhibit ferroptosis in neuronal cells by inhibiting Ptgs2, thereby attenuating traumatic brain injury (Xiao X. et al., 2019). Overall, miR-194 and miR-212-5p reduced ROS production or increased ROS decomposition by targeting key lipid peroxidation targets.

FTH1, an important subunit of ferritin, plays a crucial role in Fe^2+^ metabolism as an iron chelator (Muhoberac and Vidal, 2019). Previous studies have shown that inhibiting neurotransmitters such as glutamate A may help alleviate movement disorders (Tugan Yildiz and Tuncel Berktas, 2021). Li et al. reported that miR-335 enhances ferroptosis *in vitro* and *in vivo* models of Parkinson’s disease by degrading FTH1 (Xinrong Li et al., 2021). The pathogenesis of stroke is complex and is thought to potentially be related to glutamate-induced oxidative stress (Wu and Prentice, 2021). It was confirmed that exosomal miR-137 can inhibit oxyhemoglobin-induced ferroptosis in SH-SY5Y cells, thereby initiating protection (Li et al., 2020). Studies have shown that iron ion imbalance, oxidative stress, and abnormal glutamate have important effects on ferroptosis and nerve damage. Therefore, ferroptosis in neurological diseases is increasingly being studied. Studies have shown that the roles of microRNAs in ferroptosis of neurological diseases are involved in the complex of bidirectional regulation of neurological diseases (Wu et al., 2018). Table 3 lists microRNAs that affect neuronal injury disorder through regulating ferroptosis.

**TABLE 3:** The role of microRNA in neuronal injury disorder *via* regulating ferroptosis.

microRNA name	Target gene	Cell model	Disease name	Effect on ferroptois
miR-378a-3p	SLC7A11	HT22	Nerve injury caused by lead exposure	promote ferroptosis
miR-672–3p	FSP1	HN,PC12	Spinal Cord Injury	promote ferroptosis
miR-194	Bach1	MSCs	Oxygen-glucose deprivation/reoxygenation-induced neuronal injury	Suppress ferroptosis
miR-335	FTH1	PC12	Parkinson’s disease	promote ferroptosis
miR-137	-	SH-SY5Y	Hemorrhagic stroke	Suppress ferroptosis
miR-212–5p	Ptgs2	HT-22,Neuro-2a	Traumatic brain injury	Suppress ferroptosis

### The role of microRNA in ischemia perfusion disorders *via* regulating ferroptosis

Ferroptosis is an important mechanism leading to I/R injury in multiple organs (Chen Y. et al., 2021). GPX4 and SLC7A11 are key ferroptosis enzymes that act as target genes for multiple microRNAs in ischemic perfusion disease. Cell and animal experiments by, Zhou et al. demonstrated that miR-214–3p inhibition inhibits ferroptosis through GPX4, thereby reducing renal tubular damage (Zhou et al., 2022). Ding et al. reported that miR-182–5p and miR-378a-3p contribute to the activation of ferroptosis in I/R renal injury by inhibiting GPX4 and SLC7A11 (Ding et al., 2020). Zhang et al. showed that miR-30b-5p targets paired box protein 3 (PAX3) and SLC7A11 to enhance trophoblast ferroptosis, and that miR-30b-5p inhibition alleviates symptoms in a rat model (Zhang H. et al., 2020). GPX4 and SLC7A11 are still important microRNA target genes in ischemic perfusion disorders. This is related to GPX4’s critical antioxidant role in ferroptosis.

Steap3 is involved in ferroptosis as a key regulator of iron metabolism (Yan et al., 2021). A prior study demonstrated that miR-124–3p reduces the degree of ferroptosis in HO-1-modified bone marrow mesenchymal stem cells by inhibiting Steap3, alleviating the risk of hepatic I/R injury (Wu L. et al., 2022). Nuclear factor erythroid 2-related factor 2/heme oxygenase 1 (Nrf2/HO-1) signaling axis inhibits ROS generation and prevents oxidative stress damage (Yan et al., 2021). Tao et al. reported that miR-3587 inhibition could upregulates HO-1 by regulating HMOX1, thereby attenuating I/R-induced ferroptosis in kidney tissue (Tao et al., 2021). MiR3587 inhibition increases HO-1, a key antioxidant that reduces intracellular ROS and ferroptosis.

Xiao et al. found that miR-17–92 overexpression can affect ACSL4 expression by targeting tumor necrosis factor, alpha-induced protein 3(A20), thereby reducing ferroptosis in endothelial cells (Xiao F. J. et al., 2019). Liu et al. showed that miR-132 overexpression can accelerate the progression of atherosclerosis by reducing the level MMP, increasing ROS production, and promoting ferroptosis (Zexin et al., 2022). Ferroptosis is involved in the ischemic injury of various organs and tissues (von Samson-Himmelstjerna et al., 2022). Existing studies have shown that the mechanism through which microRNAs act on ischemic perfusion diseases mainly involve promotion of ferroptosis. Therefore, interventions may potentially target microRNA to achieve protection of target organs. Table 4 lists microRNAs that affect ischemia perfusion disorders through regulating ferroptosis.

**TABLE 4:** The role of microRNA in ischemia-reperfusion *via* regulating ferroptosis.

microRNA name	Target gene	Cell model	Disease name	Effect on ferroptois
miR-124–3p	Steap3	BMMSCs	Liver ischemia reperfusion injury	Suppress ferroptosis
miR-214–3p	GPX4	Acute kidney injury mice and Acute kidney injury	Acute kidney injury induced by cisplatin	promote ferroptosis
miR-132	_	peripheral vessels of atherosclerosis, HUVECs	Atherosclerosis	promote ferroptosis
miR-3587	HMOX1	NRK-52E	Renal ischemia-reperfusion	promote ferroptosis
miR-182–5p	GPX4	Hk-2,TCMK-1	Ischemia/reperfusion kidney injury	promote ferroptosis
miR-378a-3p	SLC7A11			
miR-30b-5p	Pax3,SLC7A11	HTR-8, TEV-1	Preeclampsia	promote ferroptosis
miR-17–92	A20	HUVEC	Endothelial cell death	Suppress ferroptosis

## Summary and outlook

**FIGURE 1:**
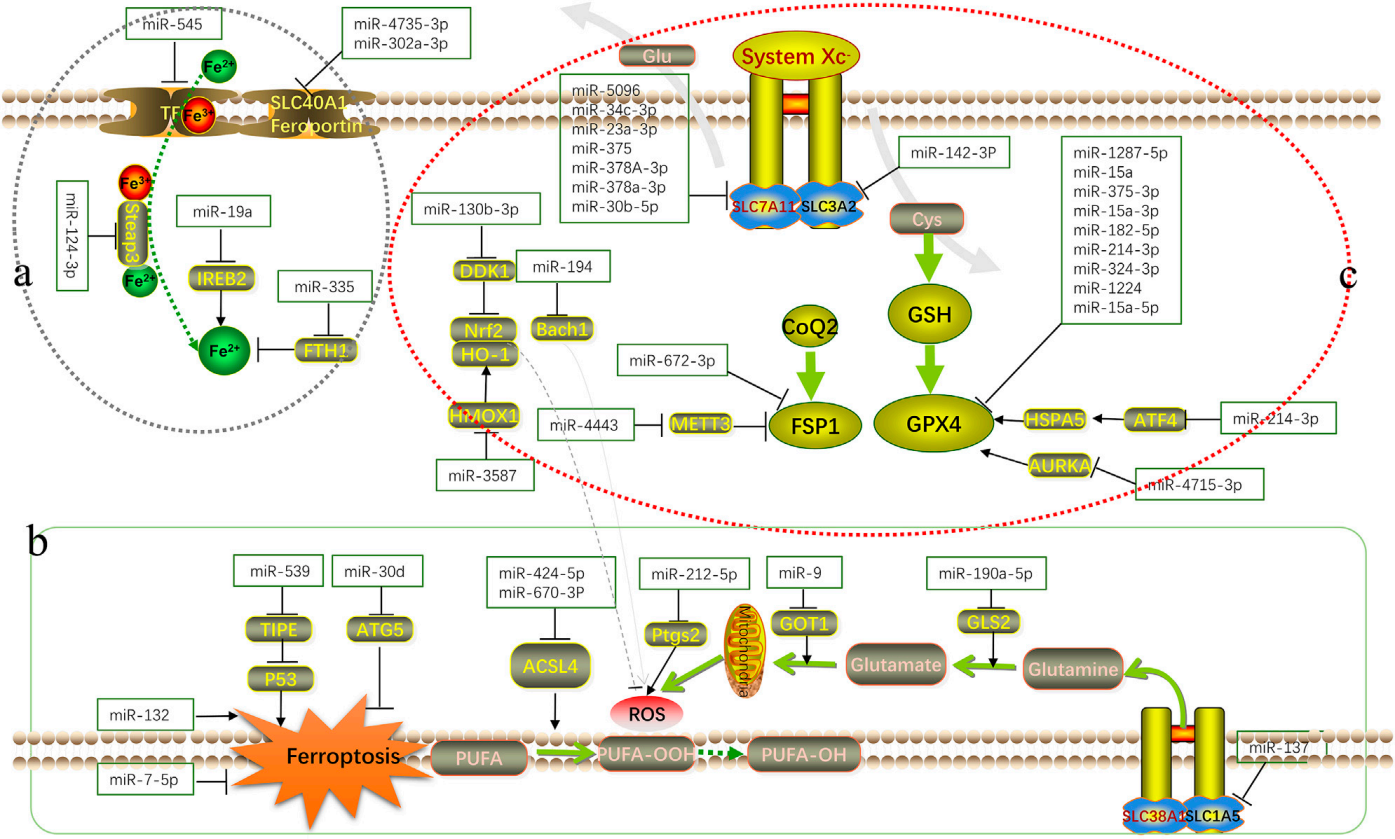
Schematic overview of the mechanism of microRNA in ferroptosis. MicroRNAs regulate various biological processes in the occurrence and development of ferroptosis by interfering with iron metabolism (**(A)**: accumulation process of intracellular Fe^2+^), lipid peroxidation (**(B)**: accumulation process of ROS and other peroxidation products) and antioxidant systems (**(C)**: antioxidant axis dominated by Xc^−^-GSH-GPX4).

Many studies have confirmed that microRNAs are involved in the occurrence of cardiomyopathy, tumors, neuronal injury disorder and ischemia perfusion disorders by promoting or inhibiting ferroptosis (Li N. et al., 2021; Maslov et al., 2022; Zuo et al., 2022). Figure 1 shows their relevant mechanisms. A significant number of studies have demonstrated that microRNAs can participate in the ferroptosis process through key targets, such as GPX4, SLC7A11, and ACSL4 (Chen et al., 2021b). Most of the current microRNA research explores its impact on a key target in the ferroptosis pathway alone, and does not explore the mechanism of action related to ferroptosis. The mechanism of microRNA’s impact on disease through regulating ferroptosis remains largely unexplored. Therefore, it is still necessary to further analyze the relevant mechanisms of microRNA from the aspects of metabolic pathways and epigenetic modifications in order to be able to, intervene in the development of diseases.
